# Calcium acts as a central player in melatonin antitumor activity in sarcoma cells

**DOI:** 10.1007/s13402-022-00674-9

**Published:** 2022-05-02

**Authors:** Ana M. Sánchez-Sánchez, María Turos-Cabal, Noelia Puente-Moncada, Federico Herrera, Carmen Rodríguez, Vanesa Martín

**Affiliations:** 1 Departamento de Morfología Y Biología Celular, Universidad de Oviedo, Asturias, Spain; 2 Instituto Universitario de Oncología del Principado de Asturias (IUOPA), , Universidad de Oviedo, Asturias, Spain; 3 Instituto de Investigación Sanitaria del Principado de Asturias (ISPA), Asturias, Spain; 4 Department of Chemistry and Biochemistry (DQB), Faculty of Sciences, University of Lisbon, Lisbon, Portugal; 5 Biosystems & Integrative Sciences Institute, Faculty of Sciences, University of Lisbon, Lisbon, Portugal

**Keywords:** Chondrosarcoma, Osteosarcoma, Melatonin, Calcium, Proliferation, Migration

## Abstract

**Purpose:**

Chondrosarcoma and osteosarcoma are the most frequently occurring bone cancers. Although surgery and chemotherapy are currently clinically applied, improved treatment options are urgently needed. Melatonin is known to inhibit cell proliferation in both tumor types. Although the underlying mechanisms are not clear yet, calcium homeostasis has been reported to be a key factor in cancer biology. Here, we set out to investigate whether regulation of calcium by this indolamine may be involved in its antitumor effect.

**Methods:**

Cell viability was measured using a MTT assay and flow cytometry was used to measure levels of cytosolic calcium, intracellular oxidants, mitochondrial membrane potential and cell cycle progression. Mitochondrial calcium was analyzed by fluorimetry. Cell migration was determined using a scratch wound-healing assay. Western blot analysis was used to assess the expression of proteins related to cell cycle progression, epithelial to mesenchymal transition (EMT), Ac-CoA synthesis and intracellular signaling pathways.

**Results:**

We found that melatonin decreases cytosolic and mitochondrial Ca^2+^ levels, intracellular oxidant levels, mitochondrial function and the expression of the E1 subunit of the pyruvate dehydrogenase complex. These changes were found to be accompanied by decreases in cell proliferation, cell migration and EMT marker expression. The addition of CaCl_2_ prevented the changes mentioned above, while co-treatment with the calcium chelator BAPTA enhanced the effects.

**Conclusions:**

Our data indicate that regulation of calcium homeostasis is a key factor in the inhibition of cell proliferation and migration by melatonin. This effect should be taken into consideration in combined therapies with traditional or new antitumor compounds, since it may circumvent therapy resistance.

## Introduction

Osteosarcoma and chondrosarcoma are the most frequently occurring bone tumors (first and second, respectively) [1]. Although current clinical treatment of osteosarcoma is based on surgery and chemotherapy and is quite effective, the side effects of the latter mean that specific and less aggressive alternative therapies continue to be sought. On the other hand, the efficacy of the treatment (60–65%) has remained unchanged in the last three-to-four decades [2]. Treatment of chondrosarcoma basically depends solely on surgery, since this type of cancer is resistant to radiation therapy and chemotherapy [3]. In addition, major resections are required in order to leave safe tumor-free margins. When these resections cannot be achieved due to the location of the tumor, curative treatment cannot be obtained. For this reason, the search for new treatment options for both types of sarcomas continues today [4].

One of the most important cellular mediators in the regulation of tumor cell proliferation, migration and metastasis is calcium homeostasis [5, 6]. Besides this, oxidative state represents a key determinant in the processes of cellular proliferation and migration, and the interrelation of these two mediators has been found to be important in the control over cancer growth [7–9]. The concentration of free radicals regulates plasma membrane voltage-gated calcium channels (VGCC) [10], exit of calcium from the cell [11], entry of calcium into the mitochondria and calcium output from cell organelles (endoplasmic reticulum -ER- or Golgi apparatus) [12]. It has also been shown that calcium regulates mitochondrial and cellular reactive oxygen species (ROS) levels, the latter mainly through the regulation of NADPH oxidases [13]. Besides its role in cell proliferation, calcium homeostasis is involved in epithelial-mesenchymal transition (EMT) [14]. EMT is involved in the formation of metastasis through losing the epithelial phenotype and increasing the expression of proteins that promote unstable junctions between epithelial cells such as N-cadherin [15], thereby being another important factor determining cancer progression.

It has amply been reported that melatonin, a hormone produced by all of the studied cell types, exhibits significant antitumor activity. Melatonin is an antioxidant molecule whose effects have been observed mainly when used at high concentrations, depending on both the regulation of antioxidant enzymes [16] and the direct scavenging of free radicals [17, 18]. Previous studies have shown that the antitumor effects of high concentrations of melatonin include the induction of cell death [19–21] and the inhibition of proliferation, migration and metastasis [22–25]. Mechanistic studies have pointed at the regulation by this indolamine of tumor metabolism with an increase in free radicals as the mechanism involved in the melatonin induction of apoptosis [26, 27]. Studies published on the inhibition of proliferation, migration and metastasis after the addition of high concentrations of melatonin point at a decrease in free radicals [24, 28] and the Akt or ERK signaling pathways, depending on the cell types involved [22, 23, 29]. Melatonin has also been found to induce EMT by regulating both E- and N-cadherin in other cancer cell types [24, 30], thereby decreasing the metastatic capacity of the tumor cells in addition to its antiproliferative effect.

Previously, regulation of cytosolic calcium by high concentrations of melatonin has been observed in non-tumor cells, regulating both calcium exit from the cell through the plasma membrane and its entry into cell organelles [31, 32]. In ovarian and colon cancer cells, melatonin has been found to decrease intracellular calcium by increasing its exit from the cell [33], while in breast cancer cells it has been found to decrease calcium currents dependent on voltage gated calcium channels [34] and to downregulate transient receptor potential cation channel 6 [35].

In the present work, the effects of high melatonin concentrations on calcium levels and the proliferation and migration of chondrosarcoma and osteosarcoma cells are studied, as well as its putative relationship with ROS regulation.

## Materials and methods

### Cell culture and reagents

MG63 (osteosarcoma) and sw-1353 (chondrosarcoma) cell lines were purchased from the American Type Culture Collection (Teddington, UK) and cultured in DMEM (Sigma- Aldrich, St Louis, MO, USA) medium supplemented with 10% v/v Fetal Bovine Serum (FBS; Gibco, Invitrogen Life Technologies, Barcelona, Spain), 100 U/ml penicillin and 100 μg/ml streptomycin (Gibco, Spain) in a humidified atmosphere of 5% CO_2_ at 37 °C. Cells were sub-cultured once a week using a 0.25% trypsin solution. Compound concentrations used for the experiments were: 1 mM melatonin, 5 µM BAPTA, 3 mM calcium chloride, 100 µM trolox and 100 µM ascorbate (Sigma-Aldrich, St Louis, MO, USA). Culture flasks and dishes were obtained from Fisher Scientific (Madrid, Spain). Fluo-3AM and Rhod-2 were purchased from Invitrogen (Invitrogen Life Technologies, Barcelona, Spain) and BAPTA/AM was acquired from Santa Cruz (Santa Cruz Biotechnology, Dallas, TX, USA). All other reagents were purchased from Sigma (Sigma-Aldrich, St Louis, MO, USA), unless otherwise indicated.

### Cell viability assay

The effect of melatonin or other compounds on cell viability was evaluated using a colorimetric assay, 3-(4, 5-dimethylthiazol-2-yl)-2,5-diphenyltetrazolium bromide (MTT). Briefly, cells were seeded in 96-well plates and once the treatments were completed, 10 µl of MTT solution in PBS (5 mg/ml) was added. After 4 h of incubation at 37 ºC, an equal volume of lysis solution [sodium dodecyl sulphate (SDS) 20% and dimethylformamide pH 4.7, 50%] was added. Next, the mixture was incubated at 37 ºC overnight and the samples were measured in an automatic microplate reader (µQuant, Bio-Tek Instruments, Inc., Winooski, VT, USA) at a wavelength of 540 nm.

### Flow cytometry

To measure cytosolic calcium levels (Fluo3-AM), intracellular oxidants (DCFH-DA) and mitochondrial membrane potential (Rhodamine 123), cells were seeded in 6-well plates and after treatment incubated with the proper fluorescence probe during 20 min at 37 °C in the dark: 1 mg/ml Fluo3-AM, 10 μM DCFH-DA in serum-free medium or 1 μg/ml rhodamine 123 in serum-free medium. Next, the cells were resuspended in 500 μl PBS and incubated with propidium iodide (final concentration 2 μg/ml) for 10 min at room temperature in the dark. Fluorescence of 10,000 live cells per group (cells negative for propidium iodide staining) was measured in a Beckman Coulter FC500 flow cytometer (Beckton Dickinson, Franklin Lakes, NJ, USA).

To analyze cell cycle progression, cells were seeded in 6-well plates and after treatment harvested and fixed in TPI overnight at 4 °C [0.0025 g propidium iodide, 50 µl Triton X-100 Detergent, 0.05 g sodium citrate in 50 ml PBS]. Next, fluorescence was measured in a Beckman Coulter FC500 flow cytometer (Beckton Dickinson Franklin Lakes, NJ, USA).

### Mitochondrial calcium level determination

Cells were seeded in 6-well plates and after treatment collected by trypsinization. Next, they were incubated with a fluorescent probe specific for mitochondrial Ca^2+^ [3 µM Rhod-2] for 30 min at 37 °C. The fluorescence signal from the cells was measured using a microplate fluorimeter FLX-800 (Bio-Tek Instruments, Inc., Winooski, VT, USA) at excitation and emission wavelengths of 552 nm and 581 nm, respectively. The obtained fluorescence was normalized to the total number of cells.

### Lactate dehydrogenase (LDH) activity assay

Cells were seeded in 24-well plates after which total LDH activity was determined following the specifications of the lactic dehydrogenase-based In Vitro Toxicology Assay Kit (Sigma-Aldrich, St Louis, MO, USA). Absorbance was determined using an automatic microplate reader (μQuant; Bio-Tek Instruments, Inc., Winooski, VT, USA) at 490 nm after which the obtained data were normalized to total protein concentrations.

### Colorimetric measurement of intracellular lactate levels

Cells were seeded in 150 mm plates after which lactate concentrations were determined using a Lactate Assay Kit according to the manufacturer's protocol (Sigma-Aldrich, St Louis, MO, USA). Absorbance was measured at 570 nm using an automatic microplate reader (μQuant; Bio-Tek Instruments, Inc., Winooski, VT, USA).

### Scratch wound-healing migration assay

To evaluate cell migration, ‘wounds’ were made in confluent 6-well plates by scratching across each monolayer using a 100 μl pipette tip. Next, the cultures were treated with melatonin, calcium chloride (3 mM final concentration) or a combination of both. Every hour an optical microscopic image of the scratched area was taken starting at time 0 h and extending to 24 h using a 10 × objective.

### Western blot analysis

For protein expression analysis, cells were seeded in 100 mm plates. After treatment, the cells were lysed with ice-cold lysis buffer (150 mM NaCl, 1 mM EDTA, 1 mM EGTA, 1% v/v Triton X-100, 2.5 mM sodium pyrophosphate, 1 mM β-glycerophosphate, 1 mM Na3VO4, 1 μg/mL leupeptin, 2 μg/ml aprotinin, 1 μg/ml pepstatin-A, 110 nM NaF, 1 mM PMSF, 20 mM Tris–HCl pH 7.5). Next, 30 μg total protein samples were separated by SDS–polyacrylamide gel electrophoresis (SDS-PAGE) and transferred to polyvinylidene difluoride membranes (Amersham Bioscience, Pittsburgh, PA, USA). The resulting blots were incubated overnight at 4 ºC with appropriate antibodies: anti-Cyclin D1 (1:1000, Cell Signaling, Danvers, MA, USA), anti-p-AKT (1:1000, Cell Signaling, Danvers, MA, USA), anti-p-ERK (1:1000, Cell Signaling, Danvers, MA, USA), anti-N-Cadherin (1:250, Santa Cruz Biotechnology, Dallas, TX, USA), anti-Vimentin (1:1000, Santa Cruz Biotechnology, Dallas, TX, USA), anti-PDH (1:500, abcam, Cambridge, UK) and anti-β-tubulin or anti-glyceraldehyde-3-phosphate dehydrogenase (GAPDH) as loading controls (1:5000, Santa Cruz Biotechnology, Dallas, TX, USA). Immunoreactive polypeptides were visualized using horseradish peroxidase conjugated secondary antibodies (anti-rabbit or anti-mousse IgG peroxidase conjugated 1:4000; Santa Cruz Biotechnology, Dallas, TX, USA) and enhanced-chemiluminescence detection reagents (Amersham Bioscience, GE Healthcare Bio-Sciences, Pittsburgh, PA, USA) following the manufacturer-supplied protocols. Western blots were analyzed using Un-Scan-It Gel 6.0 (Gel graph Digitizing Software-Silk Scientific Corporation, UT, USA) to provide quantitative values for relative expression of each protein (normalized to its own loading control).

### Statistical analysis

Results represent the average value of at least three independent experiments. Data are presented as the mean ± SEM. Significance was tested by t-test when two groups were compared, whereas one-way ANOVA followed by a Tukey test was used for multi-group comparisons. Statistical significance was accepted when *p* ≤ 0.05.

## Results

### Melatonin decreases Ca^2+^ and ROS concentrations and inhibits cell proliferation and migration

**Fig. 1:**
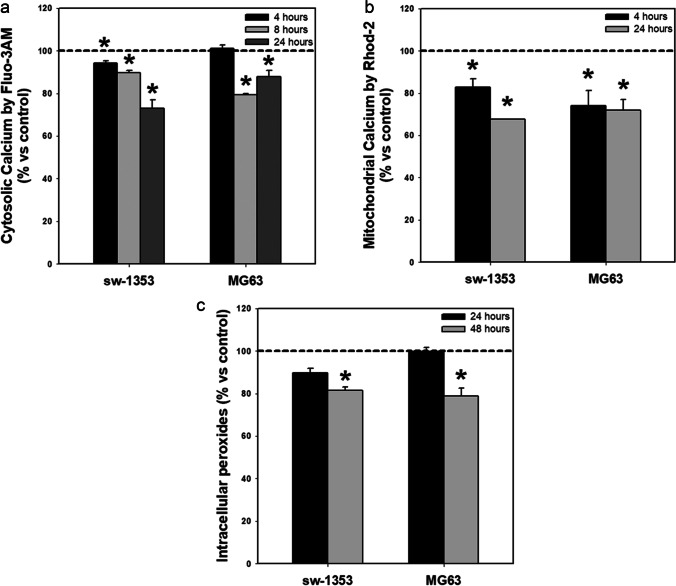
Melatonin causes decreases in cellular calcium levels and intracellular oxidants. Melatonin treatment induces a decrease in (**a**) cytosolic calcium, (**b**) mitochondrial calcium and (**c**) intracellular peroxide levels in both cancer cell lines tested. Dashed line indicates control group levels. * *p* ≤ 0.05 vs control group (vehicle-treated cells)

We found that melatonin induced a decrease in the concentration of cytosolic (Fig. 1a) and mitochondrial (Fig. 1b) Ca^2+^ in the two sarcoma cell lines tested (MG63 and sw-1353) between 4 and 24 h (Ca^2+^ levels recover after 48 h of treatment, data not shown). Intracellular ROS levels were also reduced after melatonin treatment, but this drop was only observed after 48 h of treatment (Fig. 1c).

**Fig. 2:**
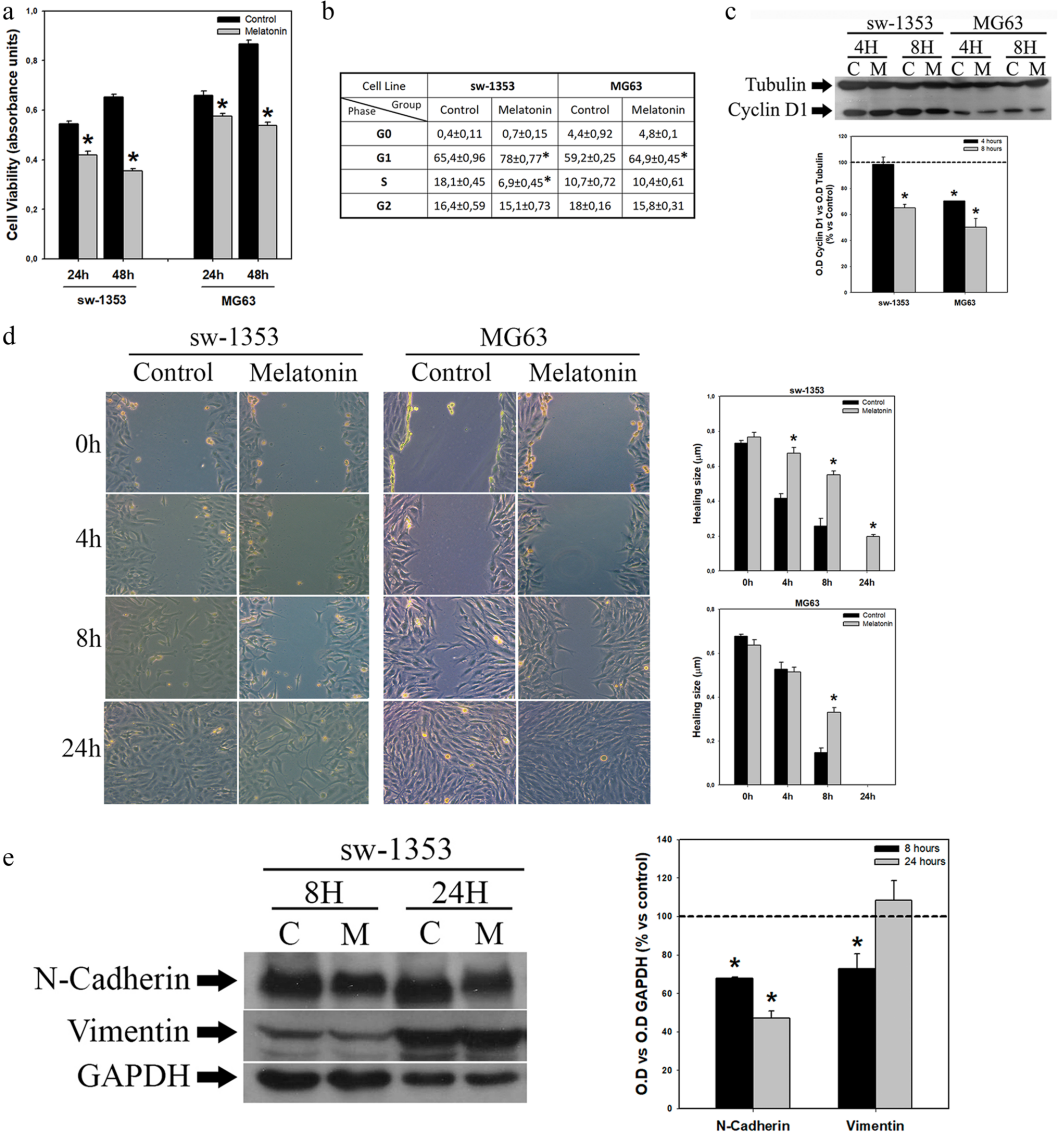
Melatonin inhibits proliferation and slows down migration of sarcoma cell lines. (**a**) Melatonin induces a reduction in the number of viable cells after 24 and 48 h of treatment. (**b**) Accumulation of cells in the G1 phase of the cell cycle observed after 24 h of treatment with melatonin. (**c**) Decreased expression of cyclin D1 induced by melatonin treatment for 4 and 8 h. Optical density reading values versus loading control tubulin are presented. Dashed line indicates control group levels. (**d**) Scratch wound-healing assay after melatonin treatment. Microscopic images taken with 10 × objective indicate that melatonin slows down cell migration. Graphs represent the mean wound healing size (µm) of each experimental group. (**e**) Decreased expression of N-cadherin and vimentin in sw-1353 cells after melatonin treatment. Optical density reading values versus loading control GAPDH are presented. Dashed line indicates control group levels. * *p* ≤ 0.05 vs control group (vehicle-treated cells)

As calcium homeostasis regulates both cell proliferation and migration, and it has been shown that melatonin regulates both processes in several types of cells, we next studied these processes in both chondrosarcoma and osteosarcoma cells. We found that changes in Ca^2+^ and ROS levels were accompanied by decreases in the numbers of viable cells after 24 and 48 h of treatment (Fig. 2a) with an increase in the number of cells in the G1 phase of the cell cycle and a decrease in the number of cells in the S phase after 24 h (Fig. 2b). The decrease in G1-S transition was confirmed by a decrease in the expression of cyclin D1 as revealed by Western blotting after 4 and 8 h of melatonin administration (Fig. 2c).

Using a scratch wound-healing assay, we found that melatonin also inhibited the migration of sarcoma cells, being more pronounced in the chondrosarcoma cells (Fig. 2d). This observation correlates well with decreases in the expression of N-cadherin and vimentin (EMT markers) after 8 h of melatonin treatment in the chondrosarcoma cells (Fig. 2e), while osteosarcoma cells showed a low basal level of N-cadherin expression (data not shown).

### Melatonin decreases mitochondrial function without changing fermentative metabolism

Mitochondrial functions such as mitochondrial respiration or ATP synthesis are also regulated by calcium homeostasis and signaling [36]. Previously, we found that melatonin decreases the expression of the E3 subunit of the pyruvate dehydrogenase complex (PDHC) in acute myeloid leukemia cells [27], which can lead to a slowdown of the Krebs cycle and, therefore, of OXPHOS. It has also been shown by others that PDHC activity / expression is regulated by Ca^2+^ concentrations [37–39]. On the other hand, a decrease in mitochondrial Ca^2+^ may be directly related to a decrease in OXPHOS [40] and, as such, may be involved in a decrease of cell proliferation, especially if it is not accompanied by an increase in fermentative metabolism that could compensate for a decrease in oxidative energy production.

**Fig. 3:**
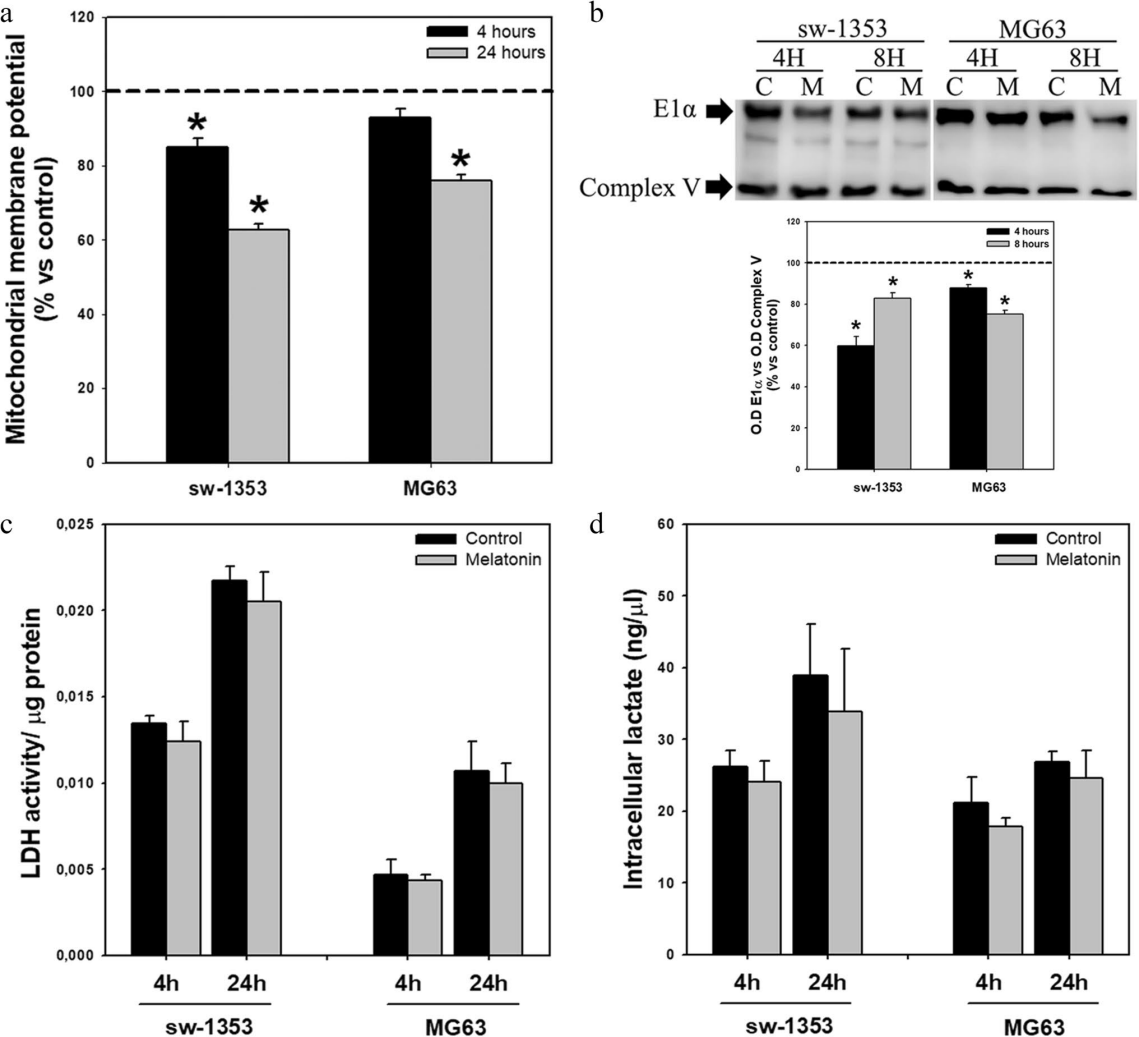
Melatonin affects mitochondrial function without modifying fermentative metabolism. (**a**) Melatonin treatment decreases mitochondrial membrane potential (ΔΨm). Dashed line indicates control group levels. (**b**) Decrease in expression of E1α subunit of the pyruvate dehydrogenase complex observed after indolamine treatment. Optical density reading values versus loading control complex V are presented. Dashed line indicates control group levels. (**c**) Melatonin does not induce changes in LDH activity or (**d**) intracellular lactate levels. * *p* ≤ 0.05 versus control group (vehicle-treated cells)

To explore whether the decrease in Ca^2+^ induced by melatonin is accompanied by changes in mitochondrial function that may be influencing cell proliferation, we first set out to study whether this indolamine modifies mitochondrial membrane potential, an indicator of mitochondrial activity [41]. We observed a decrease in mitochondrial membrane potential after 4 and 24 h of melatonin treatment (Fig. 3a). PDHC expression analysis also revealed inhibition of the expression of the E1 alpha subunit of PDHC after 4 and 8 h of treatment with the indolamine (Fig. 3b). Finally, in line with our previous results [26], no changes in fermentative metabolism, measured as LDH activity (Fig. 3c) and intracellular lactate concentration (Fig. 3d), were observed.

### Calcium homeostasis regulation is involved in the antitumor effects of melatonin

**Fig. 4:**
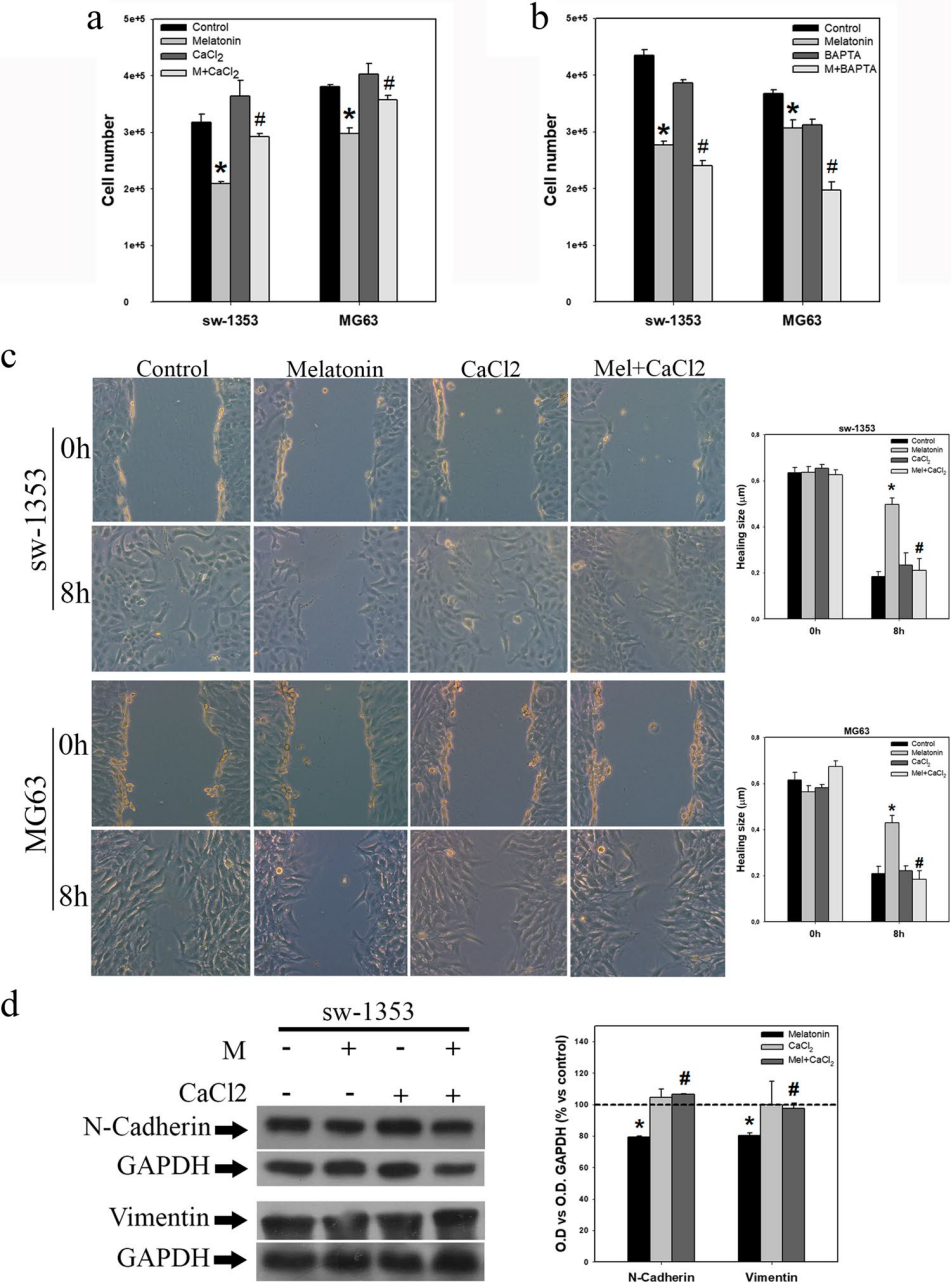
Evaluation of calcium involvement in melatonin antitumor effects. (**a**) Treatment of sarcoma cell lines with calcium chloride abolishes the antiproliferative effect induced by melatonin treatment after 48 h (**b**) while BAPTA treatment potentiates such inhibition. (**c**) Microscopic images taken with 10 × objective show that calcium chloride supplementation abolishes the effects of melatonin on cell migration. Graphs represent the mean wound healing size (µm) of each experimental group. (**d**) Combined treatment of melatonin and calcium chloride for 8 h reverses the observed melatonin effects on N-cadherin and vimentin expression in sw-1353 cells. Optical density reading values versus loading control GAPDH are presented. Dashed line indicates control group levels. * *p* ≤ 0.05 vs control group (vehicle-treated cells). # *p* ≤ 0.05 vs melatonin group

**Fig. 5:**
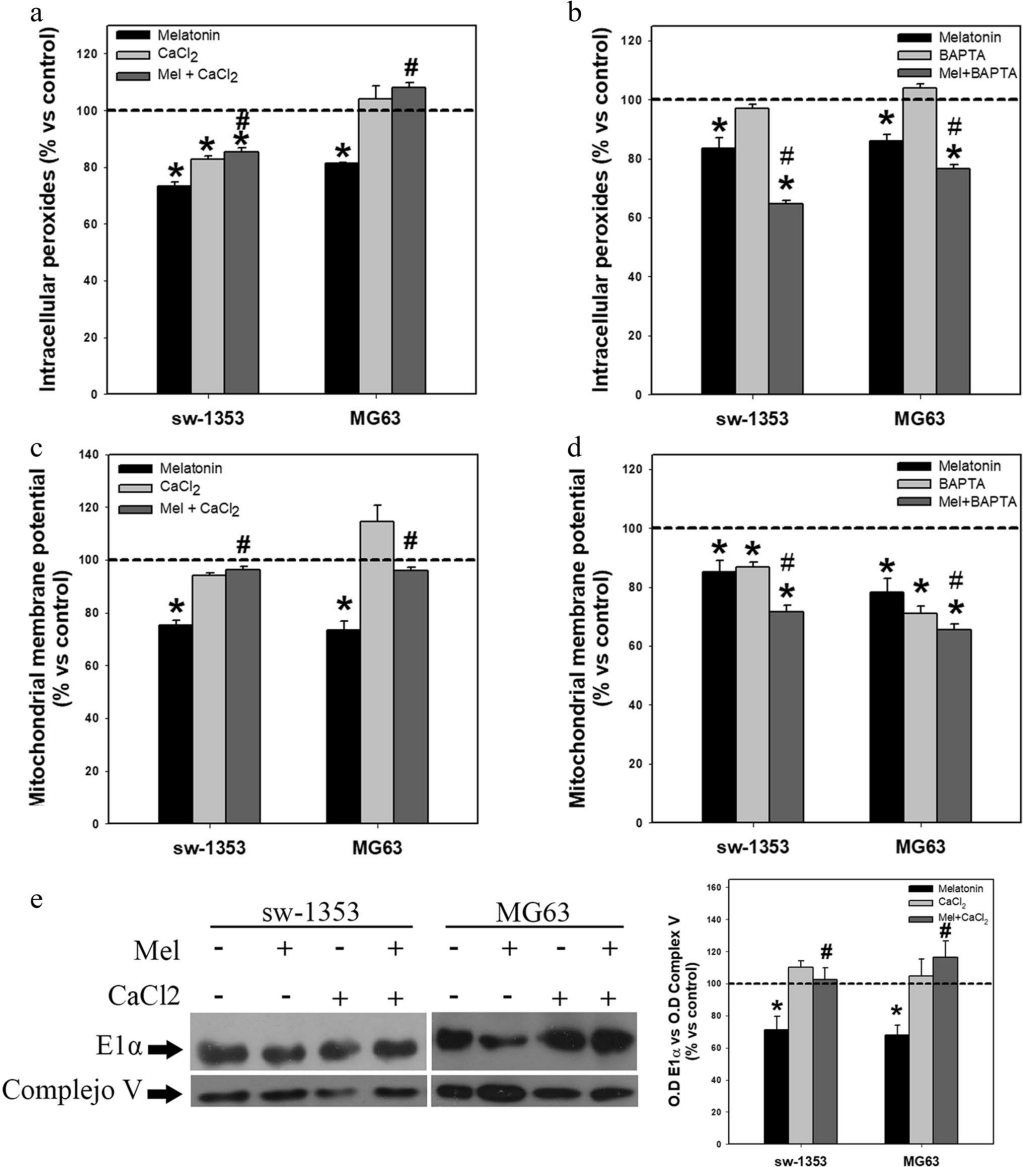
Modification of calcium levels alters melatonin effects on intracellular peroxides and mitochondrial parameters. (**a**) Calcium chloride supplementation reverses the melatonin-induced reduction in intracellular peroxide levels (48 h) while (**b**) BAPTA treatment potentiates this effect. (**c**) Calcium chloride supplementation reverses the melatonin-induced reduction in mitochondrial membrane potential (24 h) while (**d**) BAPTA treatment potentiates this effect. (**e**) Calcium chloride supplementation reverses the melatonin effect on E1α subunit expression (8 h). Optical density reading values versus loading control complex V are presented. Dashed line indicates control group levels in all graphs. * *p* ≤ 0.05 vs control group (vehicle-treated cells). # *p* ≤ 0.05 vs melatonin group

To corroborate the implication of the decrease in Ca^2+^ level in the inhibition of cell proliferation, cell migration, ROS levels and mitochondrial function induced by melatonin treatment, experiments were carried out supplementing Ca^2+^ or using Ca^2+^ chelators. We found that the addition of CaCl_2_ to the culture medium prevented almost completely the reduction in the number of cells induced by melatonin after 48 h of treatment (Fig. 4a). Conversely, we found that addition of the Ca^2+^ chelator BAPTA reinforced the antiproliferative action of the indolamine (Fig. 4b). CaCl_2_ also completely reversed the melatonin-induced inhibition of migration in both cell lines (Fig. 4c), as well as the inhibition of N-cadherin and vimentin expression induced by melatonin in chondrosarcoma cells after 8 h of treatment (Fig. 4d). Co-treatment with CaCl_2_ also prevented the decrease in cellular ROS levels after 48 h of treatment (Fig. 5a). Conversely, we found that addition of the calcium chelator BAPTA to the culture medium reinforced the decrease in ROS level induced by melatonin (Fig. 5b). The decrease in mitochondrial membrane potential after 24 h of treatment with melatonin was also abolished by the addition of CaCl_2_ (Fig. 5c), while it was reinforced by co-treatment with the calcium chelator BAPTA (Fig. 5d). Finally, we found that the inhibition of PDH E1 alpha expression after 8 h of treatment was also prevented by co-treatment with CaCl_2_ (Fig. 5e).

### MAPK kinases are not involved in the calcium-mediated effects of melatonin on cell proliferation

**Fig. 6:**
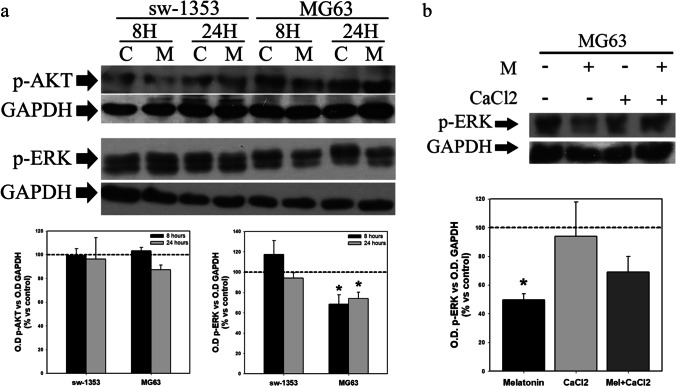
Melatonin induces a decrease in p-ERK expression in osteosarcoma cells, which is not abolished by calcium chloride. (**a**) Evaluation of the effect of melatonin p-AKT and p-ERK expression. Melatonin treatment induces a decrease in p-ERK expression in MG63 cells. (**b**) Supplementation of osteosarcoma cells with calcium chloride does not significantly abolish the decrease in p-ERK expression caused by melatonin after 24 h of treatment. Optical density reading values versus loading control GAPDH are presented. Dashed line indicates control group levels in all graphs. * *p* ≤ 0.05 vs control group (vehicle-treated cells). # *p* ≤ 0.05 vs melatonin group

The decrease in Ca^2+^ observed after melatonin administration may be due to the regulation by this indolamine of different calcium channels, either those located in the plasma membrane or in the ER. Activation of Ca^2+^ channels induces intracellular signaling cascades mediated by different pathways, such as the PI3K/Akt, MAPK, calcitonin or calcineurin pathways. Of these, the PI3K/Akt pathway has been implicated in intracellular signaling after activation of IP3R channels, while ERK is involved in signaling after activation of voltage-gated Ca^2+^ channels of the plasma membrane [42]. The expression of phosphorylated Akt and ERK as determined by Western blotting was assessed in chondro- and osteosarcoma cells after treatment with melatonin. We found that this treatment inhibited ERK phosphorylation in osteosarcoma cells after 8 and 24 h, while it had no effect on Akt activation. No effect of melatonin was observed neither on Akt nor in ERK activation in chondrosarcoma cells (Fig. 6a). The addition of CaCl_2_ did not significantly abolish the inhibition of ERK activation by melatonin in osteosarcoma cells (Fig. 6b).

### ROS regulation by melatonin is not involved in intracellular Ca^2+^ concentration and mitochondrial function

**Fig. 7:**
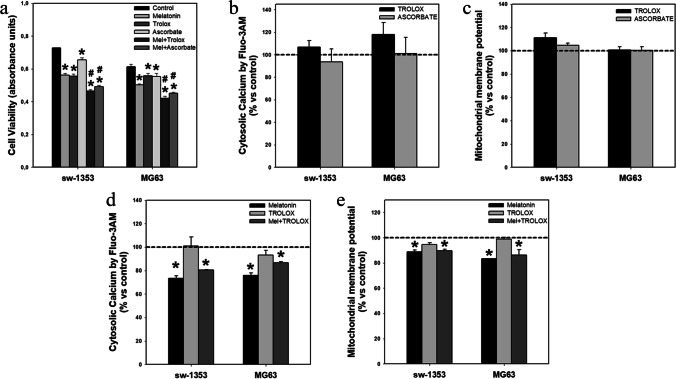
Antioxidants enhance melatonin-induced inhibition of cell viability, but do not affect intracellular calcium levels or mitochondrial membrane potential. (**a**) Trolox and ascorbate potentiate melatonin effects on the number of viable cells after 48 h of treatment. (**b**) Treatment with trolox or ascorbate for 24 h does not modify cytosolic calcium levels or (**c**) mitochondrial membrane potential. (**d**) Combined treatment for 24 h of melatonin with Trolox does not modify the effect of the indolamine on cytosolic calcium levels or (**e**) mitochondrial membrane potential. Dashed line indicates control group levels in graphs b-e. * *p* ≤ 0.05 vs control group (vehicle-treated cells). # *p* ≤ 0.05 vs melatonin group

To study the interrelationship between the antioxidant effect of melatonin and its regulation of calcium concentration in the two human sarcoma cancer cell lines, comparative experiments were carried out using two other antioxidants. We found that the addition of antioxidants to the culture medium did not have the same effect as melatonin. Although antioxidants potentiated the antiproliferative effect of melatonin (Fig. 7a), this potentiation was independent of the regulation of Ca^2+^ levels by these molecules, since there was no effect on cytosolic calcium concentration after 24 h of treatment (Fig. 7b). Twenty-four hours of treatment with antioxidants also failed to decrease the mitochondrial membrane potential (Fig. 7c). Finally, co-treatment with trolox did not reinforce the effect of melatonin, neither on the cytosolic calcium concentration nor on the mitochondrial function (Figs.7d and e).

## Discussion

It is well known that tumor cells exhibit a constitutive increase in global Ca^2+^ concentration [43], being involved in the regulation of e.g. cell proliferation, migration and metastasis [42]. Genetic alterations in tumor cells and changes in the microenvironment that surrounds them may cause activation of different calcium channels and pumps that induce calcium increase inside the cell and activate various functions such as cell proliferation and migration [44]. Regulation by melatonin of different Ca^2+^ channels and pumps [31–35, 45] may explain the downregulation of Ca^2+^ levels by this indolamine as observed in our present work, as well as its prevention of the increase in cytoplasmic Ca^2+^ induced by factors reported by others [46, 47]. In fact, Ca^2+^ is necessary for cell cycle progression, in particular for passage from the G1 to the S phase or from the G2 to the M phase, being a key regulator of cellular growth [48]. Consistently, we found in the cell lines studied here, that after melatonin treatment there was an increase in cells in the G1 phase and a decrease in cells in the other phases of the cell cycle, indicating a slowdown in the passage from G1 to S. Moreover, addition of calcium to the culture medium abolished the effects of melatonin on cell proliferation, while addition of a calcium chelator potentiated such effects.

Activation or overexpression of calcium channels may directly regulate intracellular signaling pathways, involving different MAP kinases, thereby increasing cell proliferation [42]. The association of Ca^2+^ to binding proteins such as calmodulin (CAM) also activates calmodulin-dependent kinases (CAMK), which in turn are necessary for the activation of cyclin-dependent kinases (CDKs) [48], and can activate the ERK pathway [5] and transcription factors necessary for cyclin synthesis [49]. An involvement of various signaling pathways, such as the PI3K/Akt/mTOR [23] or the ERK [29] pathways in the regulation of cell proliferation by melatonin has also been reported. We did not observe inhibition of the Akt pathway after melatonin treatment in either cell line, but partial inhibition of ERK was noted in osteosarcoma cells. However, this inhibition was not abolished by the addition of calcium to the culture medium. It seems, therefore, that inhibition of proteins in the G1-S transition, and so of cell proliferation, mediated by the decrease of calcium after melatonin administration is not dependent on these MAP kinases, neither in sw-1353 nor in MG63 cells. It is possible that the decrease in Ca^2+^ concentration itself, through its lower binding to calcium-binding proteins, downregulates the enzymes and/or transcription factors involved in cell cycle progression. We can, however, not rule out that the decrease in ERK after melatonin administration may contribute to inhibition of MG63 cell proliferation.

The observation that addition of CaCl_2_ abolished the decrease in mitochondrial function induced by melatonin, while co-incubation of this indolamine with the calcium chelator BAPTA potentiated the decrease in mitochondrial function, suggests an involvement of calcium regulation by melatonin in this parameter. A decrease in mitochondrial Ca^2+^ may cause a reduced mitochondrial function either directly, depending on the concentration of mitochondrial Ca^2+^ itself [36, 40] or indirectly through regulation of the pyruvate dehydrogenase complex (PDHC), which determines the availability of NADPH for mitochondrial respiration. This complex is dependent on Ca^2+^, both for its activation [39, 50, 51] and for the expression of dihidrolipoamide dehydrogenase (DLD) and pyruvate dehydrogenase (PDH), the E3 and E1 proteins of the complex, respectively [37, 38]. In the present work, a decrease in PDH expression was observed after the addition of melatonin, which was abolished by the addition of calcium to the culture medium. This indicates that regulation of the calcium concentration by melatonin may be responsible for the down-regulation of PDH expression, which in turn may, at least partially, be responsible for the decrease in mitochondrial function and consequently, sarcoma cell proliferation.

A decrease in free cytoplasmic Ca^2+^ can also affect cell migration and EMT, which are key to metastasis formation. Cadherins belong to one of the main families of molecules involved in EMT, migration and metastasis. Of these, the most studied in cancer are E-cadherin and N-cadherin, with opposite roles in relation to migration: E-cadherin promotes stable cell adhesion and N-cadherin promotes unstable adhesions, which facilitate migration. It has been shown that tumors expressing N-cadherin are more aggressive, even though they retain E-cadherin expression [52, 53], and that an increase in N-cadherin expression promotes migration in several cancer cell types [52, 54–56]. In the present study, we found that melatonin inhibited cell migration, particularly in chondrosarcoma cells. Retarded cell migration was accompanied by decreases in the expression of N-cadherin and vimentin in chondrosarcoma cells, and the addition of Ca^2+^ abolished both retarded cell migration and decreased N-cadherin expression. The osteosarcoma cells tested showed a very low basal expression of N-cadherin and a slight inhibition of migration by melatonin. Differences in basal N-cadherin expression levels between the two tumor types may explain the weaker inhibition of migration by melatonin. A relationship between the concentration of intracellular Ca^2+^ and the expression of N-cadherin has previously been reported in tumor [57] and non-tumor cells [58]. Also, inhibition of EMT by melatonin has previously been reported [24, 30], although the underlying mechanisms were not clarified.

Although reactive oxygen species (ROS) can regulate various calcium channels and pumps, it has been found that also Ca^2+^ can regulate ROS production [13]. Given that melatonin has antioxidant effects [16, 17], it remains to be established whether the decrease in ROS induced by melatonin regulates calcium channels, whether the regulation of calcium channels by melatonin contributes to the decrease in the concentration of ROS or whether they are independent effects contributing to a decrease in cell proliferation after treatment with this indolamine. Our data indicate that antioxidants have similar effects to melatonin in terms of inhibiting cell proliferation. However, they do not affect the decrease in Ca^2+^ concentration induced by this indolamine, which suggests that both effects may contribute independently to the inhibition of cell proliferation and possibly to the inhibition of EMT.

Tumor cells are known to exhibit alterations in calcium transport and signaling systems [59]. These alterations give tumor cells an advantage with respect to survival and proliferation and, therefore, control of calcium signaling has been proposed as a new objective in the fight against resistance to current antitumor therapies [48]. In this sense, it has previously been shown that melatonin can potentiate various chemotherapeutic agents in different tumor types, although the underlying mechanisms have not been elucidated [60]. Regulation of calcium levels by this indolamine should be taken into consideration in the use of combined therapies with traditional or novel antitumor drugs, since this may be instrumental in avoiding resistance to these compounds.

## Data Availability

Not applicable.
